# Treating hippocampal neural stem cells with nano-pulsed laser therapy generates neurons resilient against amyloid-β oligomer toxicity

**DOI:** 10.1177/13872877261416086

**Published:** 2026-01-28

**Authors:** Kevin Johnson, Auston Grant, Shrinath Kadamangudi, Armando Arizpe, Kathia Johnson, Rinat Esenaliev, Giulio Taglialatela, Maria-Adelaide Micci

**Affiliations:** 1 Department of Anesthesiology, The University of Texas Medical Branch at Galveston, Galveston, TX, USA; 2 The Mitchell Center for Neurodegenerative Disorders, Department of Neurology, The University of Texas Medical Branch at Galveston, Galveston, TX, USA; 3 Department of Neurobiology, The University of Texas Medical Branch at Galveston, Galveston, TX, USA; 4 Moody Brain Health Institute, The University of Texas Medical Branch at Galveston, Galveston, TX, USA

**Keywords:** Alzheimer's disease, amyloid-β, low-level laser therapy, neural stem cells, neurogenesis

## Abstract

**Background:**

Hippocampal synaptic dysfunction driven by toxic amyloid-β oligomers (AβO) is an early event in the progression of neurodegeneration and cognitive decline in Alzheimer's disease (AD). Non-invasive photobiomodulation therapy (PBM) is a promising intervention that has been shown to reduce amyloid and tau pathology, improve synaptic function, and preserve hippocampal neurogenesis in animal models of AD. Nano-pulsed laser therapy (NPLT) is a type of PBM therapy using pulsed 808 nm near-infrared laser light and optoacoustically generated ultrasound waves to stimulate deeper brain structures than would be accessible by traditional PBM therapy. We hypothesize that NPLT can effectively modulate hippocampal neurogenesis to induce resilience against AD.

**Objective:**

To assess resilience of hippocampal neurons derived from NPLT-treated neural stem cells (NSC) against AβO toxicity.

**Methods:**

We use NPLT to stimulate adult hippocampal neural stem cells (NSC) then induce neuronal differentiation in vitro and assess the mature neurons for AβO binding capacity and mitochondrial toxicity, and gene expression changes after NPLT.

**Results:**

We found that neurons differentiated from NPLT-treated NSC are resilient against AβO binding and mitochondrial toxicity, and show increased expression of genes associated with autophagy and proteostasis.

**Conclusions:**

Our findings support the hypothesis that NPLT modulation of hippocampal neurogenesis can be an effective non-invasive approach to induce resilience against AD toxic oligomers.

## Introduction

Neurodegenerative diseases are among the most financially and emotionally taxing conditions in the modern world. Alzheimer's disease (AD), the most common form of dementia, is the 7^th^ leading cause of death in the United States and the only one of the top ten to have increased more than 140% in the last twenty years.^1^ This dramatic increase in mortality indicates the lack of significant progress in therapeutic development despite advances in our broader understanding of the disease. One of the many criticisms of the past decades of AD research is the hyper-focused approach of eliminating the hallmark pathological protein aggregates, amyloid-β plaques and tau neurofibrillary tangles, to try to cure the disease. The recent reports from the Donanemab and Lecanemab monoclonal anti-amyloid antibody trials suggest that this approach could be effective for slowing the progression of dementia symptoms especially if implemented early but also made clear that this approach alone would not be sufficient to reverse or cure the disease.^2,3^ It has been well documented that the insidious onset of AD pathology occurs for decades prior to the emergence of clinical symptoms^4,5^ at which point cortical and hippocampal atrophy have crossed the threshold of cognitive reserve in the affected individual. For this reason, it is critical to investigate alternative therapeutic interventions that can address cellular processes that have long since broken down by the time dementia symptoms emerge. Harnessing the therapeutic potential of adult hippocampal neurogenesis is one possible strategy to address the underlying pathology of AD and help restore cognitive function in those afflicted by the disease.

For nearly a century neuroscience was dominated by a central dogma that in the adult brain, neurons and the networks they compose were fixed, immutable, and incapable of regeneration.^6,7^ In the 1960s Joseph Altman discovered DNA synthesis, thus, cell division, occurring in the hippocampal dentate gyrus of adult rats;^8,9^ however, it was another decade later that Kaplan and colleagues confirmed the newly divided cells were in fact neurons.^10^ Despite these discoveries, adult neurogenesis and its proponents were largely written off as quacks until the 1990s, when neural stem cells were isolated from mammalian brains and demonstrated proliferation and multipotency to differentiate into neuronal and glial cell types in vitro.^11,12^ Today, adult hippocampal neurogenesis (AHN) is a widely accepted phenomenon and its implications in aging, learning, and memory are actively under investigation.

Studies have shown that normal aging slows hippocampal neurogenesis, which is in part due to age-related metabolic decline,^13–16^ and this process is significantly accelerated in the presence of AD neuropathology, leading to depletion of both neural stem cells (NSC) and newborn neurons.^17–19^ Photobiomodulation (PBM) using red to near-infrared light is an accessible, non-invasive therapy known to reduce inflammation and regulate cell metabolism via stimulation of mitochondrial cytochrome c oxidase (CCO).^20–25^ In human clinical trials, transcranial PBM has shown therapeutic benefits for AD, traumatic brain injury (TBI), and mood disorders.^26–30^ In addition to its metabolic and anti-inflammatory effects, PBM improved hippocampal neurogenesis in animal models of AD, TBI, and stroke^31–35^

The efficacy of PBM is dependent on three principal variables: wavelength of light, fluence or energy density of the treatment, and whether it is delivered as continuous or pulsed wave. Red light in the 650–700 nm wavelength range, and near-infrared light (NIR) at 800–850 nm align with the peak absorption values of CCO and are the most commonly used wavelengths in PBM therapy.^22,31,36^ Unlike visible red light, which is largely reflected and scattered by the surface of the skin, the longer wavelength NIR penetrates much deeper through skin, enhancing therapeutic benefits to underlying tissues.^37,38^ Fluence, or energy density, is used to calculate the “dose” of PBM therapy delivered and is typically measured in J/cm^2^. Because mitochondrial respiration generates oxygen free radicals (ROS), important signaling molecules in low concentrations, but cytotoxic at high concentrations, there is an optimal therapeutic window above which the therapeutic benefits can be lost and potential negative effects introduced.^39^ Finally, delivery of PBM as continuous or pulsed wave light can have differential effects, as pulsed wave light has a built in cool down period during the light “off” cycle it allows for delivery of a higher power, thus higher energy at greater tissue depth, without the detrimental tissue heating effects found with continuous wave delivery of the same power.^40,41^

We proposed nano-pulsed laser therapy (NPLT) utilizing short (nanosecond) light pulses to generate optoacoustic waves in tissues and demonstrated therapeutic effects of NPLT in animal models of TBI.^42,43^ In this study, we used NPLT to treat adult hippocampal neural stem cells in vitro and induced their differentiation into mature neurons. We found that these neurons retain a resilient phenotype against amyloid-β oligomers long after NPLT treatment, and exhibit enhanced protein synthesis and autophagy-related gene expression. These results suggest that photobiomodulation of NSC in the hippocampal neurogenic niche can produce new neurons resilient to proteotoxic stress in AD and other neurodegenerative disorders.

## Methods

### Neural stem cell culture and mature neuron differentiation

Adult female rat hippocampal neural stem cells (NSC; Millipore) were cultured as neurospheres according to the supplier's protocol. Briefly, cells were grown in NSC expansion media composed of 50:50 Dulbecco's Modified Eagle Medium (DMEM)/F12 supplemented with 2% B-27, 20 ng/mL FGF, and 1% antimycotic/antibiotic. For experimentation on mature neurons, cells were differentiated according to the same supplier's protocol. NSC were seeded on laminin-coated 8-well chamber slides or 6-well plates for 24 h in NSC expansion media to allow for attachment, then switched to neuronal differentiation media containing 50:50 DMEM/F12, 2% B-27 supplement, 1% antimycotic/antibiotic, 1 μM retinoic acid, and 5 μM Forskolin for five days, changing media every other day. This protocol results in a differentiated population of approximately 70% mature neurons (MN), and 30% glia or undifferentiated stem cells. For quality control, NSC and differentiatied cells are checked via western blot and immunocytochemistry for the stemness markers SOX2 or Nestin, MN marker βIII-tubulin, and the astroglial marker GFAP (Supplemental Figure 1).

### In vitro NPLT treatment

**Figure 1.**
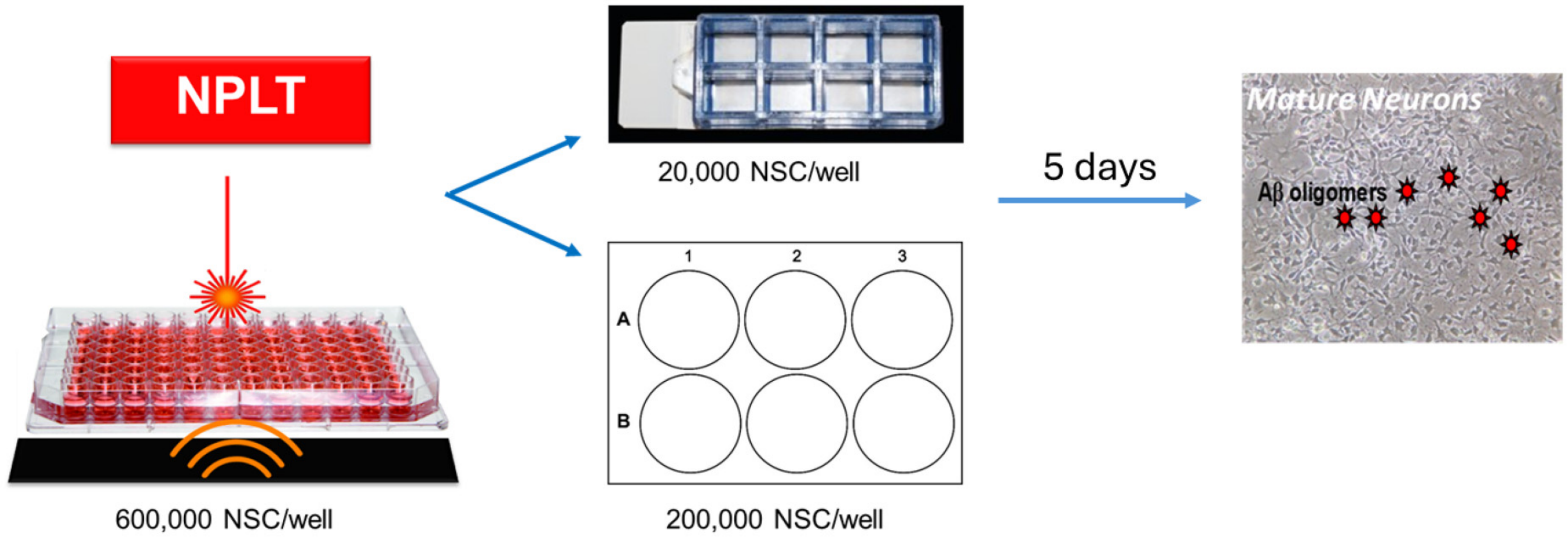
Experimental design. Schematic showing NPLT treatment of hippocampal NSC followed by 2.5 μM Aβ oligomer exposure.

NSC were dissociated using Accutase (Millipore-Sigma) for accurate counting, and 600,000 NSC were added to a single well of a 96-well plate. To simulate optoacoustic ultrasound generation that occurs by thermoelastic expansion of tissue during NPLT treatment,^44^ a photoabsorbent black membrane was adhered to the base of the well using ultrasound gel. The purpose of the membrane is to completely absorb light energy and reflect only optoacoustic ultrasound waves back into the well. Cells were placed beneath the 3 mm diameter laser probe with approximately 14 mm from the tip of the probe to the bottom of the well, resulting in a laser spot of 0.11 cm^2^ and treated for approximately 5 min with 808 nm NPLT (20 Hz, ∼5 mJ/pulse, 10 ns pulse width) for a total dose of ∼300 J/cm^2^ (Figure 1). Sham-treated NSC were plated in the same density for the same duration but not exposed to the NPLT device. Immediately following NPLT or Sham treatment, NSC were seeded on laminin-coated chamber slides or plates for differentiation.

### Amyloid-β oligomer preparation

Synthetic monomeric Aβ_1–42_ peptide was purchased from AnaSpec (cat# AS-GMP-20276-1; Fremont, CA). Aβ oligomer (AβO) preparation was performed as previously described.^45–47^ Briefly, 0.3 mg of lyophilized Aβ peptide was dissolved in 200 µl of 1,1,1,33,3-hexafluoro-2-propanol (HFP) and incubated at room temperature for 10–20 min followed by dilution with 700 µl of ultrapure water, and the mixture was magnetically stirred in a fume hood for 48 h at room temperature (RT). To facilitate the evaporation of HFP, a cap with four holes was placed on the tube for the duration of stirring. The resulting AβO were aliquoted, frozen at −80°C, and used within 3 months of preparation. For immunofluorescence analysis of AβO binding to cultured neurons, the HFP-Aβ peptide solution was spiked with HyLite Fluor 647-tagged monomeric Aβ (cat# AS-64161; AnaSpec Inc., Fremont, CA, USA) then oligomerization proceeded as described resulting in fluorescent 647-AβO. The quality of the oligomeric preparation was verified by western blot analysis using the AβO-specific 6E10 antibody (cat# 803002; BioLegend, San Diego, CA, USA). The characterization and toxicity of AβO prepared by this method has been thoroughly documented by our group and others.^46,48,49,50^

### Amyloid-β oligomer binding to mature neurons

To determine whether mature neurons derived from NPLT-treated NSC (NPLT-MN) are resilient to AβO binding, we treated NSC with NPLT or Sham treatment as described above, then differentiated the cells on laminin-coated 8-well chamber slides for five days at a density of 50,000 cells/well. On the fifth day of differentiation, when the majority of cells express the mature neuron marker βIII-tubulin, we exposed NPLT- and Sham-MN to 2.5 μM 647-AβO diluted in fresh differentiation media, and incubated cells at 37°C for 30 min. After 30 min cells were washed three times with Dulbecco's phosphate-buffered saline (DPBS), followed by 30-min fixation with 4% paraformaldehyde at room temperature. Fixed cells were then washed three times with DPBS and immunostained for the neuronal cytoskeletal protein βIII-tubulin (cat# G712A; Promega, Madison, WI), and the nuclear stain DAPI. Slides were then imaged on an Olympus FV1200 laser scanning confocal microscope at 60× magnification. Images were manually quantified by a blinded experimenter using imageJ to measure total neurite length, and total neurite-bound 647-AβO puncta. A minimum of 50 neurites were quantified per well, data are represented as single-well averages for Sham-MN (n = 10 wells) and NPLT-MN (n = 11).

### Mitochondrial membrane potential assay

Mitochondrial dysfunction is a known phenotype in AD-affected neurons, and Aβ has been shown to directly impair mitochondrial transport and respiration.^51–53^ Photobiomodulation (PBM) with near-infrared light (NIR), including 808 nm light used in NPLT, is also known to produce therapeutic benefits by stimulation of mitochondrial respiration via cytochrome c oxidase.^22,54^

To test metabolic resilience against AβO in NPLT-MN we used a flow cytometry-based Mitotracker assay using the mitochondrial membrane potential-dependent dye Mitotracker Deep Red (MTDR; cat# M22426; Thermo Fisher, St Louis, MO) to label actively respiring mitochondria. This technique has been demonstrated to detect changes in mitochondrial membrane potential induced by direct and indirect inhibition of mitochondrial electron transport^55,56^ Sham- and NPLT-MN cultures were incubated for 30 min with 2.5 µM AβO in complete differentiation media in 6-well plates. After incubation, cells were washed with fresh media then replaced in media containing 15 nM MTDR and incubated another 30 min. After Mitotracker staining cells were rinsed once with DPBS then detached using Accutase (Millipore, Burlington, MA). Detached cells were collected and spun down for 1 min at 300xG. Cell pellets were resuspended in 500 µL DPBS and kept on ice for flow cytometry analysis. MTDR fluorescence was measured using the red (642 nm) laser in a Guava EasyCyte 8 flow cytometer with GuavaSoft InCyte software (Millipore, Burlington, MA), a total of 15,000 events were acquired per sample. Acquisition gates were established based on FSC versus MTDR fluorescence values for control samples of unlabeled cells (Supplemental Figure 2). Data are represented as the % of cells within the MTDR + gate.

### mRNA sequencing and western blot assay

**Figure 2.**
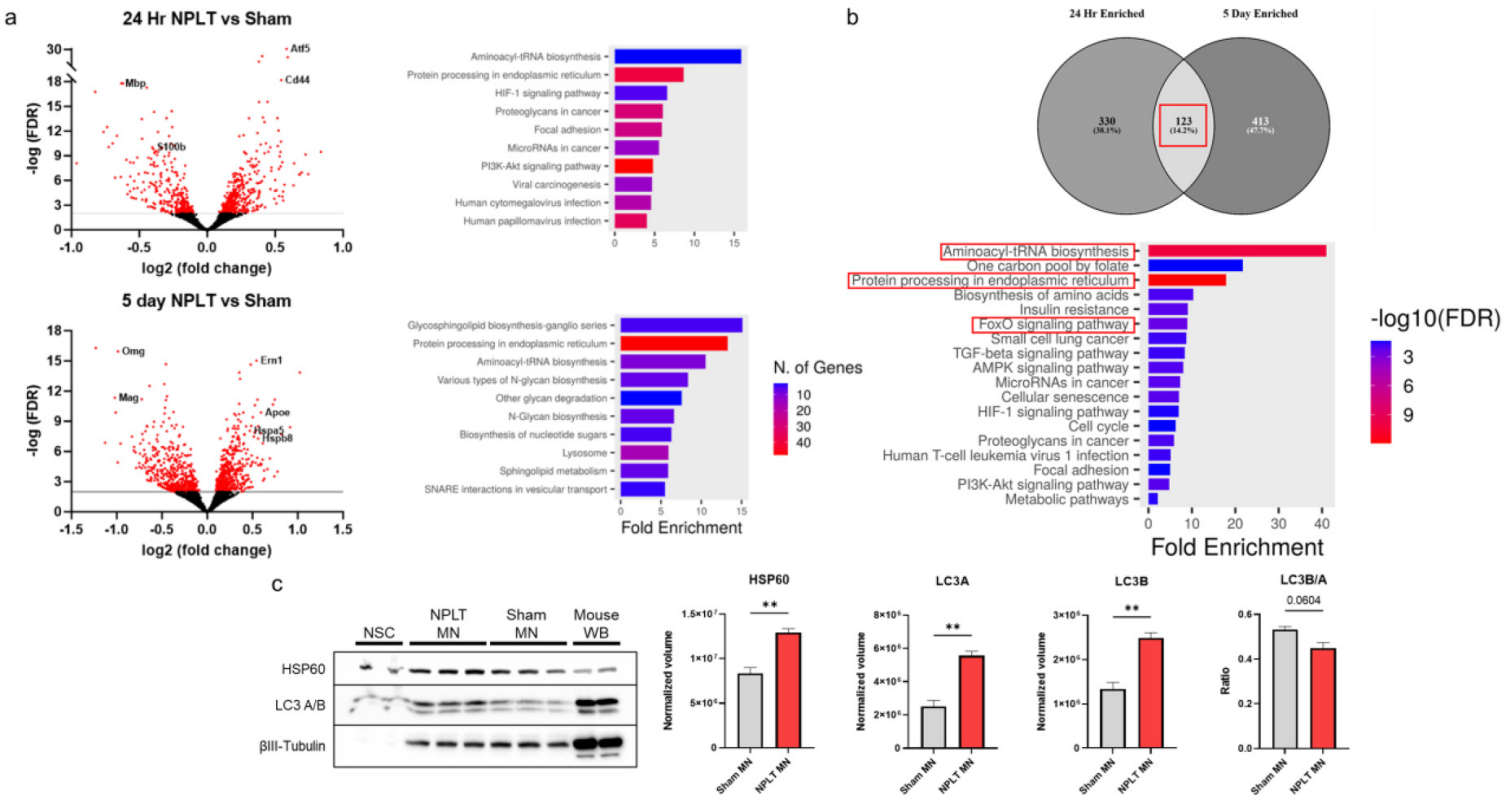
NPLT enhances autophagy-related gene expression in NSC and mature neurons derived from NPLT-treated NSC. (a) Volcano plots and GO pathway analysis for enriched genes in NPLT-NSC 24 h after treatment and after 5 days of neuronal differentiation in vitro. (b) 123 genes enriched both in NSC at 24 h and mature neurons after differentiation map to pathways involved in protein synthesis, processing, and autophagy. (c) Western blot analysis of proteins extracted from mature neurons (MN) differentiated from sham- or NPLT-treated NSC shows increased expression of autophagy related proteins HSP60 and LC3 (A and B). (Unpaired t-test with Welch's correction, **p < 0.01). NSC and mouse whole brain tissues were used as antibody controls.

To investigate potential mechanisms for induced resilience in NPLT-MN, we treated NSC in 96-well plates with ∼300 J/cm^2^ NPLT then seeded them on laminin-coated 6-well plates at a density of ∼20,000 NSC/cm^2^ and conducted bulk RNA sequencing and western blot analysis. Cells were collected either at 24 h for assessment of early gene expression changes in NSC, or induced toward neuronal differentiation for 5 days, then collected for RNA or protein isolation. Gene expression analysis of NPLT-NSC was conducted by UTMB's Next Generation Sequencing core facility. Briefly, RNA was isolated from triplicate sham- and NPLT-treated NSC 24-h after treatment, or from mature neurons after a 5-day differentiation using the Direct-Zol RNA miniprep kit (Zymo research) following the manufacturer's protocol. RNA samples were then forwarded to the Next Generation Sequencing core facility for library preparation using the NEBNext Ultra II RNA library for illumina sequencing kit (New England Biolabs# E7775) combined with NEBNext polyA mRNA magnetic module (Catalog# E7490). The sequencing was performed on the NextSeq 550 (Illumina) using High Output 150 cycle kit for PE 75 sequencing. Reads were mapped using DESEQ2 and differentially expressed genes in NPLT compared to Sham were determined using an adjusted p value cutoff of 0.01. Pathway analysis of enriched genes was conducted using ShinyGO V0.81.^57^ Differentially expressed genes were compared between time points using Venny.^58^ A subset of cells from the 5-day differentiation group were separately collected and lysed using radio immunoprecipitation assay (RIPA) buffer plus HALT protease inhibitor cocktail (Thermo Fisher) for protein extraction. Protein concentration was quantified using a Pierce BCA assay kit (Thermo Fisher) and proteins were separated by gel electrophoresis using Novex tris-glycine 10–20% gels. Separated proteins were transferred to PVDF membrane and blotted using the following primary antibodies: HSP60 (Abcam ab46798), LC3A/B (Cell signaling 4108S), and βIII-tubulin (Abcam ab18207). Appropriate HRP-conjugated secondary antibodies were applied before chemiluminescent visualization using SuperSignal West Pico Plus ECL substrate (Thermo Fisher). Target protein bands were normalized to βIII-tubulin (Figure 2(c)).

## Results

### NPLT-MN are functionally resilient to Aβo toxicity

**Figure 3.**
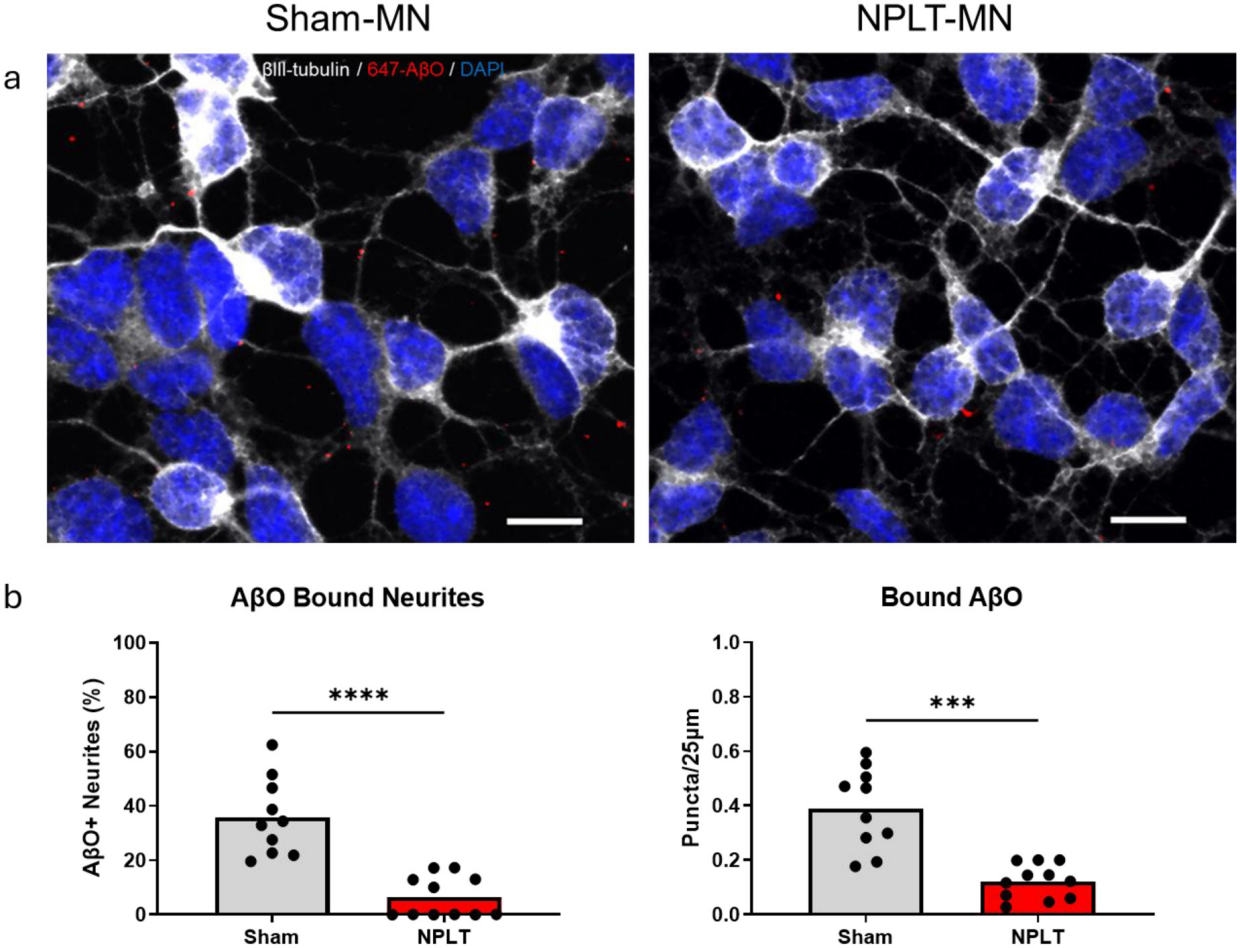
NPLT-MN show reduced binding of 647-labeled AβO to neurites. **(**a) Representative confocal microscope images of mature neurons bound with fluorescent-labeled AβO (scale bar = 10 μm). (b) Quantifications of the total fraction of neurites with bound AβO (left) and the total number of AβO bound per 25 μm of neurites quantified. Welch's t-test ***p < 0.001, ****p < 0.0001.

To assess resilience of NPLT-MN against Aβ oligomer binding, we incubated cells with HiLyte Fluor-647-labeled AβO and quantified 647-AβO puncta bound to neuritic projections (Figure 3). Our analysis revealed that NPLT-MN had significantly fewer neurites with bound 647-AβO compared to Sham-MN (6.44 ± 7.58% versus 35.83 ± 14.10), and fewer 647-AβO puncta when normalized per 25 μm of neurite length (0.12 ± 0.06 versus 0.39 ± 0.15; Figure 3(b)). We found no differences in the average neurite length between NPLT-MN and Sham-MN (25.91 ± 2.52 μm versus 23.24 ± 3.74).

**Figure 4.**
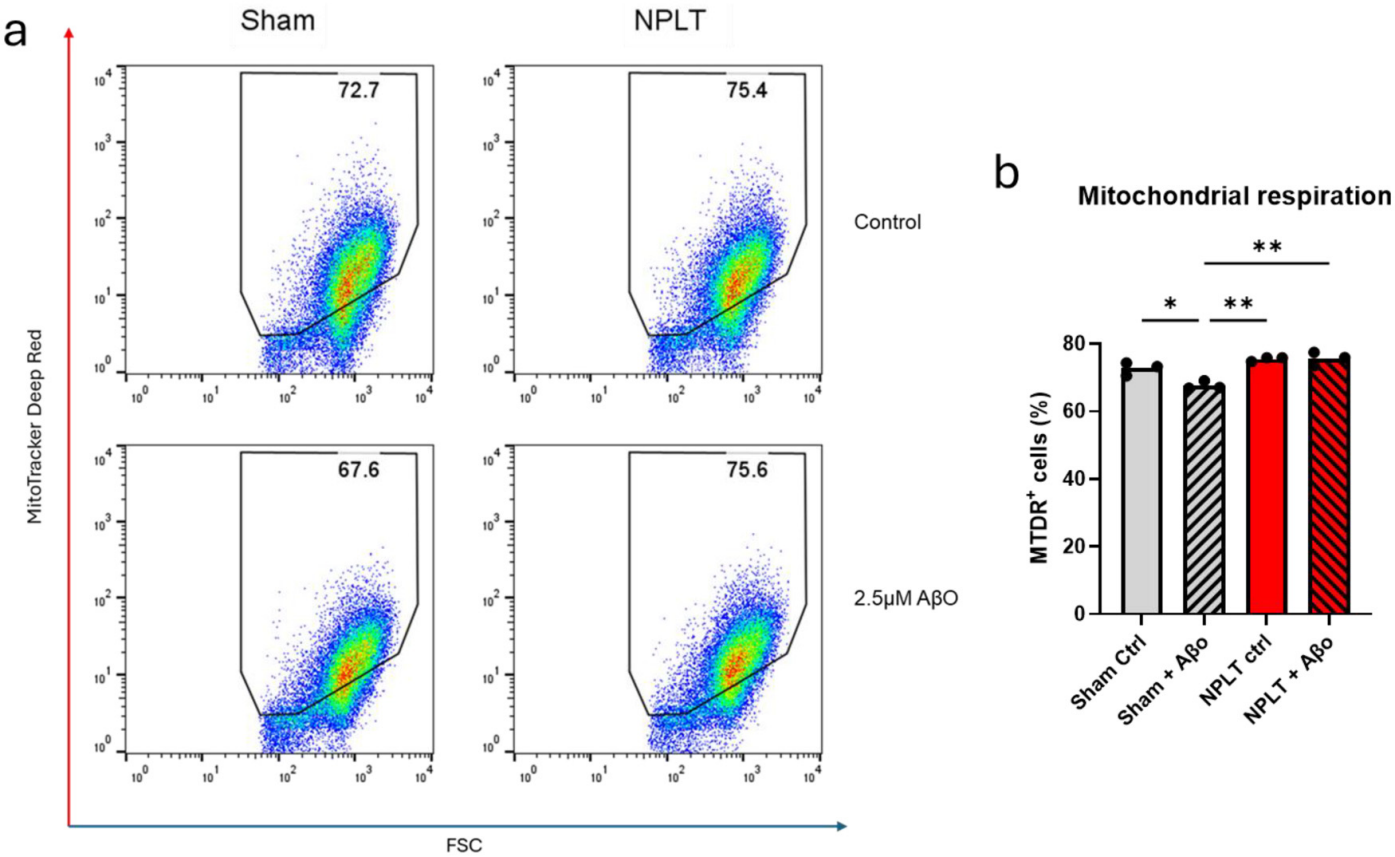
NPLT-MN are resilient to AβO-induced mitochondrial toxicity. (a) Scatterplots showing Mitotracker Deep Red fluorescence Sham- and NPLT-MN with and without 30-min exposure to 2.5 μM AβO. (b) Quantification of MTDR + cell populations from each treatment condition. One-way ANOVA *p > 0.05, **p < 0.01.

We next tested whether functional resilience accompanied the decreased binding of AβO to NPLT-MN using a well-established Mitotracker assay to quantify active mitochondrial respiration after AβO exposure. Our results show that AβO exposure caused a significant decrease in the number of metabolically active cells in Sham-MN, but not NPLT-MN. Similarly, AβO-treated NPLT-MN had a significantly higher portion of metabolically active cells compared to AβO-treated Sham-MN (75.60 ± 1.97% versus 67.60 ± 1.22; Figure 4(b)).

### NPLT modulates autophagy-related gene expression in cultured NSC and their progeny

To investigate potential mechanisms of induced resilience in NPLT-MN, we treated NSC with NPLT and collected RNA for next-gen sequencing at 24 h post treatment before inducing differentiation, and after 5 days of differentiation. In total, 453 genes were significantly upregulated and 322 downregulated in NPLT-NSC compared to sham after 24 h, and 536 upregulated versus 461 downregulated after neuronal differentiation. Pathway analysis at each time point shows upregulation of genes involved in protein biosynthesis and processing and lipid membrane biosynthesis (Figure 2(a)). Comparison of upregulated gene expression between NSC at 24 h and MN after 5-day differentiation showed 123 genes in common, and pathway analysis of these genes indicated sustained upregulation of genes involved in protein synthesis and processing as well as the FoxO signaling pathway which is a known activator of autophagy and a key regulator of cell metabolism and survival (Figure 2(b)). Western blotting of Sham- and NPLT-MN showed a significant upregulation of the chaperone protein HSP60 and the autophagosome-associated proteins LC3A and LC3B in NPLT MN, consistent with the persistent upregulation of p62 expression found in both RNAseq datasets (Figure 2(c)). These results suggest enhanced autophagy and maintenance of protein folding stress are associated with sustained resilience of mature neurons generated from NPLT-treated NSC *in vitro.*

## Discussion

Modulation of adult hippocampal neurogenesis is a promising potential therapeutic strategy for the treatment of AD and other neurodegenerative diseases. It is well documented that AHN declines in normal aging, and to a greater extent in AD.^14,16,18,59–62^ Despite some evidence suggesting that soluble Aβ species can promote neural stem cell proliferation and neurogenesis, these changes are often found to deplete the NSC pool and yield incomplete neuronal maturation and increased gliogenesis.^63–67^ Both the preservation of the NSC pool in the hippocampal dentate gyrus, and the stimulation of neurogenesis from hippocampal NSC have produced cognitive and neuropathological benefits in animal models.^35,46,68–70^ Non-invasive transcranial PBM has been shown to modulate hippocampal neurogenesis in TBI, stroke, and AD.^31–35^^,43^ Therapies specifically targeting AHN could prove extremely beneficial for restoring cognitive function in AD, considering the significant role newborn granule neurons play in hippocampal plasticity.^71–73^ However, even in pre-clinical stages of AD, soluble Aβ and tau oligomers threaten the survival of vulnerable newborn neurons,^64,74^ therefore, it is imperative that AHN targeted therapies also promote the survival and integration of newborn neurons without also depleting the NSC pool. Here we show, for the first time, acquired resilience in mature neurons differentiated from adult hippocampal NSC treated with PBM.

Soluble Aβ oligomers are believed to be the earliest forming toxic species in the canonical progression of AD, and have been shown to cause synaptic dysfunction, mitochondrial dysfunction, and impaired neurogenesis long before the deposition of amyloid plaques and neurofibrillary tangles.^48,51–53^^,64,75–78^ It has also recently been shown that Aβ oligomer binding at synapses can facilitate internalization of toxic tau oligomers, leading to neurodegeneration.^79^ PBM effectively induces synaptic resilience against Aβ and tau oligomer toxicity in animal models of AD;^48,80^ however, to our knowledge this is the first demonstration of Aβ-resilient progeny derived from hippocampal neural stem cells treated with PBM. Using a well-established model of preformed labeled Aβ oligomers, and the neuron-specific marker βIII-tubulin, we show reduced AβO binding to neurites, and fewer total neurites affected by AβO in mature neurons derived from NPLT-treated NSC compared to Sham. In addition, NPLT-MN were resilient against metabolic toxicity induced by AβO as shown by our mitochondrial respiration assay. To establish a potential mechanism for the acquired resilience phenotype found in NPLT-MN, we conducted transcriptomic analysis by bulk RNA sequencing both NSC 24 h after NPLT treatment, and MN after a full 5-day differentiation. The sequencing results show an immediate shift towards enhanced pro-neuronal gene expression and decreased glial gene expression as well as enrichment in pathways associated with protein biosynthesis and the misfolded protein response, both essential processes in the differentiation of stem cells into neurons.^13,81^ In our mature neuron population, we again found enrichment of genes associated with the misfolded protein response as well as lipid biosynthesis and processing, including Apolipoprotein E, whose function is directly linked to clearance of Aβ and mitochondrial function.^82–86^ When comparing the two time points it became evident that autophagy, a critical process for metabolic and protein folding homeostasis, was a likely contributor to the sustained resilience phenotype. Autophagy is correlated with resilience against AD neuropathology in cognitively intact individuals^87^ and has been shown to increase with PBM in animal models of AD.^80^ In neural stem cells, autophagy pathways under the control of the transcription factor FOXO3 maintain proteostasis,^15^ but upon differentiation into neurons, proteostasis becomes largely dependent on chaperone proteins such as the small heat shock protein HSP60.^88^ HSP60 is of particular interest due to its ability to be secreted extracellularly and bind to AβO, altering its conformation and reducing toxicity.^50,89^ It is possible that increased expression of HSP60 in NPLT-MN facilitates neutralization and sequestration of AβO prior to AβO engaging synapses, preventing synaptic and metabolic dysfunction. Alternatively, NPLT may enhance cell-autonomous protein processing pathways, leading to improved ability to maintain homeostasis during the significant protein folding stress of neuronal differentiation, leading to broadly healthier mature neurons that can resist AβO toxicity.

A known phenomenon in PBM therapy is the biphasic dose response in which therapeutic efficacy peaks at a specific energy density, above which effects are lost or altogether reversed. This effect is thought to be mediated by reactive oxygen species (ROS).^39^ Under homeostatic conditions ROS act as signaling molecules to regulate metabolic pathways, cell proliferation and survival, and inflammation,^23,24,90,91^ but under conditions of metabolic stress excessive ROS can oxidize proteins, lipids, and nucleic acids leading to DNA damage and cell death.^92^ While we did not directly measure ROS production in this study, we can make inferences about the effects of NPLT on ROS production from our 24 h RNAseq dataset. In addition to protein synthesis and processing, NPLT-NSC showed increased expression of genes involved in hypoxia-inducible factor 1 (HIF-1) and phosphatidylinositol 3-kinase-Akt (PI3K-Akt) signaling pathways, both of which are activated by ROS to induce metabolic reprogramming, cell proliferation and survival.^91^ Notably, DNA damage repair pathways are overrepresented in the most downregulated genes of that dataset, and we found no evidence of NF-κB activation or any other inflammatory response suggesting that NPLT-NSC effectively maintain redox homeostasis during proliferation and differentiation without the harmful effects of excessive ROS production. PBM has been shown to modulate additional neuronal resilience pathways such as PKA/Sirt1, Creb, and BDNF in vivo;^32,34,42,93^ however, we did not see any evidence of changes to these pathways in our model.

Further research is necessary to determine the extent of the acquired resilience phenotype of NPLT-MN in vivo because in the era of medicine where cell replacement therapies are already becoming a reality for some neurodegenerative diseases,^94–97^ the ability to generate new, resilient neurons could significantly improve long-term patient outcomes.

Presently available Aβ-targeted therapies for AD show some effectiveness for slowing the progression of cognitive decline; however, to date, we have no way to effectively restore cognitive function with AD therapies.^2,3,98,99^ Non-invasive PBM could provide an accessible means to restore or improve cognitive function by stimulating AHN, allowing the formation and integration of new synaptic connections in the hippocampus, and ensuring the survival of newly formed connections between resilient neurons.

## Supplemental Material

sj-docx-1-alz-10.1177_13872877261416086 - Supplemental material for Treating hippocampal neural stem cells with nano-pulsed laser therapy generates neurons resilient against amyloid-β oligomer toxicity Supplemental material, sj-docx-1-alz-10.1177_13872877261416086 for Treating hippocampal neural stem cells with nano-pulsed laser therapy generates neurons resilient against amyloid-β oligomer toxicity by Kevin Johnson, Auston Grant, Shrinath Kadamangudi, Armando Arizpe, Kathia Johnson, Rinat Esenaliev, Giulio Taglialatela and Maria-Adelaide Micci in Journal of Alzheimer's Disease
