# Supplementary material for: Treating hippocampal neural stem cells with nano-pulsed laser therapy generates neurons resilient against amyloid-β oligomer toxicity
Source: J Alzheimers Dis. 2026 Jan 28;110(2):705–15. doi: 10.1177/13872877261416086 (PMC13066474; doi:10.1177/13872877261416086)
Supplement: sj-docx-1-alz-10.1177_13872877261416086 - Supplemental material for Treating hippocampal neural stem cells with nano-pulsed laser therapy generates neurons resilient against amyloid-β oligomer toxicity [file sj-docx-1-alz-10.1177_13872877261416086.docx]

**Supplemental Material**

**Treating hippocampal neural stem cells with nano-pulsed laser therapy generates neurons resilient against amyloid-β oligomer toxicity**


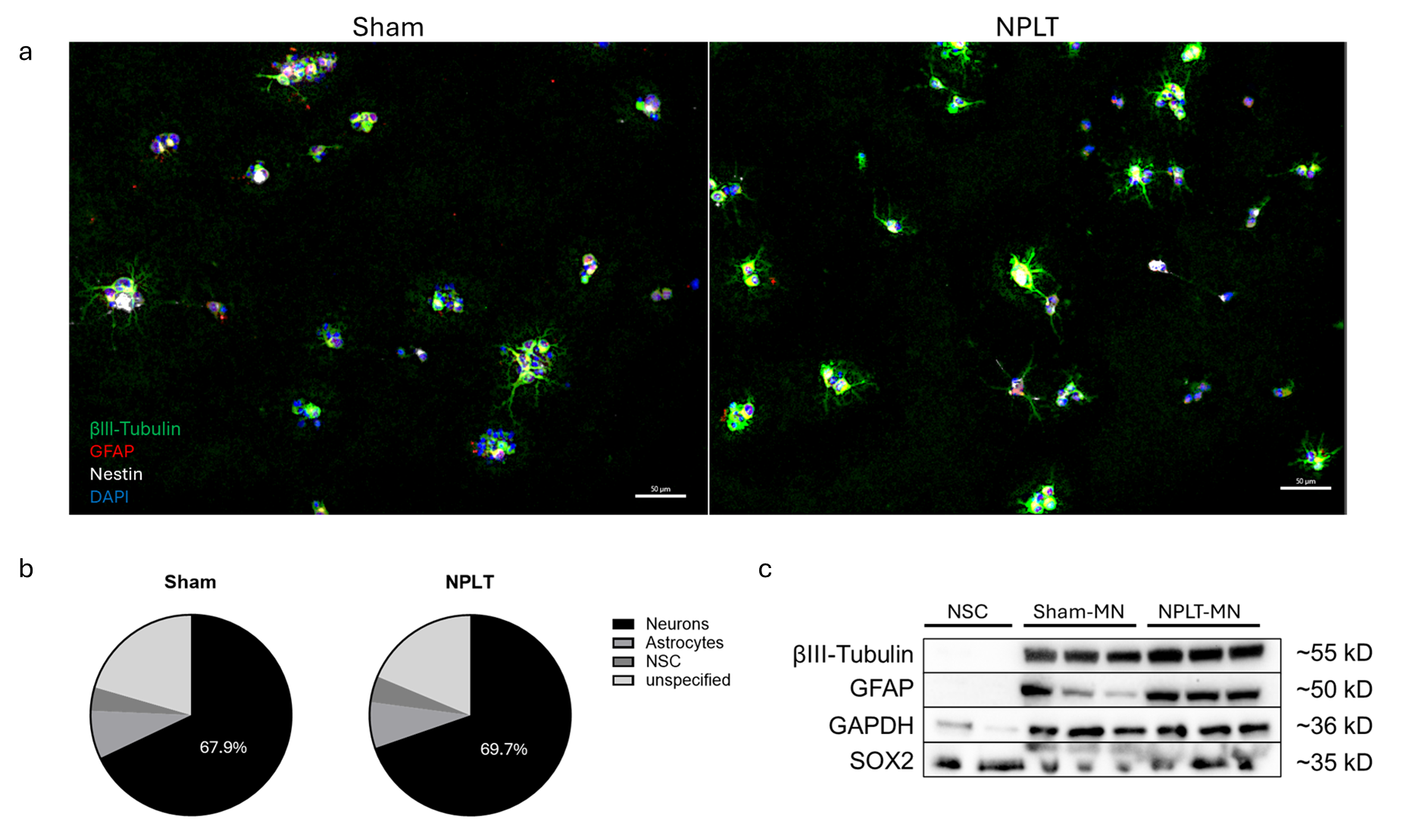


**Supplemental Figure 1. Quality control for NSC and differentiated cells. (**A) Representative images of post-differentiation cells stained for the MN marker βIII-Tubulin, glial marker GFAP, and stem cell marker Nestin. (B) Quantification of cell type distribution in differentiated cultures from sham and NPLT-NSC. (C) Characterization of NSC and differentiated cultures by western blot for βIII-Tubulin, GFAP, GAPDH, and SOX2.


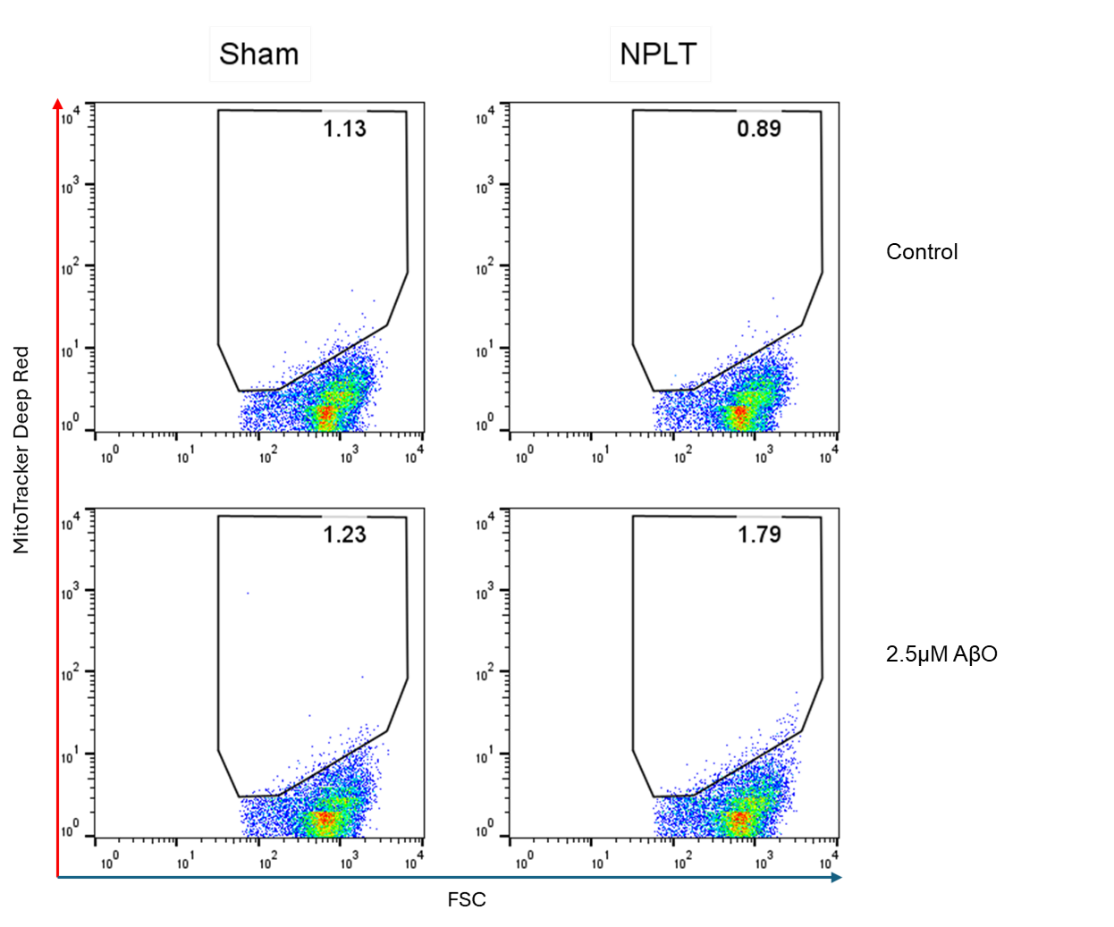


**Supplemental Figure 2. Flow cytometry controls.** Gating controls for flow cytometry experiments were established using cells exposed to each experimental condition without the addition of MTDR.
